# Identifying Immune Cells as Mediators in the Impact of Gut Microbiota on Congenital Malformations of the Nervous System

**DOI:** 10.1002/brb3.71150

**Published:** 2025-12-31

**Authors:** Haokun Tian, Xiaolan Guo, Jialu Yu, Yuanfang Lu, Wensen Cao, Xiuwei Wang, Zhiqiang Zhu, Zhen Guan, Aiyun Yang, Shen Li, Xiaochun Xu, Caihua Wang, Jianhua Wang

**Affiliations:** ^1^ Laboratory of Translational Medicine, Beijing Municipal Key Laboratory of Child Development and Nutriomics Capital Institute of Pediatrics Beijing China; ^2^ Graduate School of Peking Union Medical College Chinese Academy of Medical Sciences Beijing China; ^3^ Beijing Key Laboratory of Environmental and Viral Oncology, Faculty of Chemistry and Life Sciences Beijing University of Technology Beijing China; ^4^ Guanghua Academy Shanghai China; ^5^ Fenyang People's Hospital Fenyang Shanxi China

**Keywords:** congenital malformations, gut microbiota, immune cells, Mendelian randomization, nervous system, neural tube defect

## Abstract

**Introduction:**

Congenital malformations of the nervous system, such as neural tube defects, are significant contributors to infant morbidity and mortality. This study aimed to assess the causal influence of gut microbiota on nervous system malformations and to identify immune cells as potential mediators in this relationship, utilizing a two‐step Mendelian randomization (TSMR) approach.

**Methods:**

Using genome‐wide association studies data on gut microbiota, immune cells, and congenital malformations of the nervous system, we applied TSMR to evaluate the total, direct, and mediating effects. Gut microbiota indicators served as exposures, congenital malformations of the nervous system as outcomes, and immune cell markers as mediators. Statistical analyses included MR Egger, inverse variance weighted, and so forth.

**Results:**

Our analysis identified 19 gut microbiota indicators causally associated with congenital malformations of the nervous system. Notably, *Eubacteriaceae* and *Eubacterium* genera exhibited protective effects, while *Escherichia* and *Bacteroides* genera showed positive correlations with malformation risk. Nine immune cell indicators significantly mediated these effects. For example, CD20^+^ B cells were negatively associated with malformation risk, suggesting a protective immune response, whereas CD25^+^ B cells were positively correlated, indicating increased malformation risk.

**Conclusions:**

This study demonstrated a significant link between gut microbiota composition and congenital nervous system malformations, mediated by specific immune cells. These findings highlighted the potential for gut microbiota modulation and immune‐targeted therapies as preventive or therapeutic strategies in reducing malformation risks. Future investigations should aim to replicate these results across diverse populations and further elucidate the underlying biological mechanisms of gut–immune–neural interactions.

**Trial Registration:**

The authors have nothing to report.

## Graphical abstract

This study reveals causal links between gut microbiota and congenital nervous system malformations, identifying nine immune cell types, such as CD20+ and CD25+ B cells, as mediators, highlighting gut–immune interactions as potential preventive or therapeutic targets.

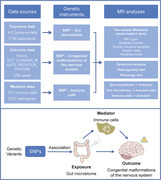

## 1 Introduction

Congenital malformations of the nervous system represent a significant category of birth defects that are associated with severe clinical implications, often leading to developmental disabilities, impaired neurological function, and, in some cases, early mortality (Tian et al. 2024). These malformations encompass a broad range of structural anomalies, including neural tube defects (NTDs), brain and spinal cord malformations, and other abnormalities affecting the central and peripheral nervous systems (Cruz et al. 2021; Dias and Partington 2015; Wang et al. 2024). They contribute substantially to infant morbidity and mortality rates worldwide, presenting a critical challenge for public health and clinical care.

The etiologies of nervous system malformations are complex and multifactorial, often involving genetic and environmental factors that influence neural development during early embryogenesis (Avagliano et al. 2019; X. Wang et al. 2023; Yue et al. 2021). Traditional studies have focused on genetic mutations, maternal health conditions, nutritional deficiencies, and exposure to environmental toxins as potential risk factors. However, recent advances in genomics, immunology, and microbiology have highlighted an emerging area of interest—the role of the gut microbiota and immune system in modulating neural development and the risk of congenital abnormalities (Cordero‐Varela et al. 2023; Denny et al. 2013). The gut microbiota, a diverse community of microorganisms residing in the human gastrointestinal tract, is increasingly recognized as a critical regulator of immune function and systemic inflammation (Rendeli et al. 2023). It communicates with the central nervous system through what is known as the “gut–brain axis,” impacting neurodevelopmental processes in complex ways (Socała et al. 2021).

Emerging studies now suggest that alterations in gut microbiota composition may contribute to developmental defects, including those affecting the nervous system, by modulating immune responses (Cordero‐Varela et al. 2023; Denny et al. 2013). Recent experimental and clinical evidence supported a gut–immune–placenta–fetus pathway that plausibly linked maternal microbiota to fetal neurodevelopment. Microbial‐derived metabolites and molecular patterns (e.g., short‐chain fatty acids, tryptophan metabolites, and lipopolysaccharide) could modulate maternal systemic and local immune responses, leading to downstream effects on placental function, fetal neuroinflammation, microglial development, and neuronal differentiation during critical windows of embryogenesis (Loh et al. 2024; O'Riordan et al. 2025). This biological rationale provided a framework for interpreting Mendelian randomization (MR) results that implicated immune cell phenotypes as mediators between gut microbiota and congenital nervous system malformations. The immune system, which serves as an interface between gut microbiota and host physiology, is hypothesized to mediate the influence of microbial populations on neurodevelopmental outcomes via “gut–immune–brain axis.” Thus, a deeper understanding of these interactions will reveal novel insights into the mechanisms underlying congenital nervous system malformations and inform preventive or therapeutic strategies targeting microbiota–immune interactions in at‐risk populations.

MR serves as a powerful approach for causal inference by leveraging genetic variants as instrumental variables to minimize confounding common in observational studies. By using randomly assorted genetic variants associated with exposures (e.g., gut microbiota and immune cells), MR can more accurately infer causality (Richmond and Davey Smith 2022). This approach is especially suitable for investigating the causal relationship between gut microbiota, immune cells, and congenital nervous system malformations. Through a two‐step MR (TSMR) analysis, both direct and immune cell‐mediated effects of gut microbiota on neural development will be identified, offering a robust framework to dissect these complex interactions (Q. Wang et al. 2023).

This study aims to determine the causal influence of gut microbiota on congenital nervous system malformations and to identify immune cells as potential mediators. By clarifying the role of immune cells in this pathway, the research contributes to understanding the mechanisms of neurodevelopmental defects. These insights could inform preventive and therapeutic approaches, potentially guiding interventions that target gut microbiota and immune modulation in populations at risk, thereby supporting better neurodevelopmental outcomes.

## 2 Methods

### 2.1 Study Design

**FIGURE 1:**
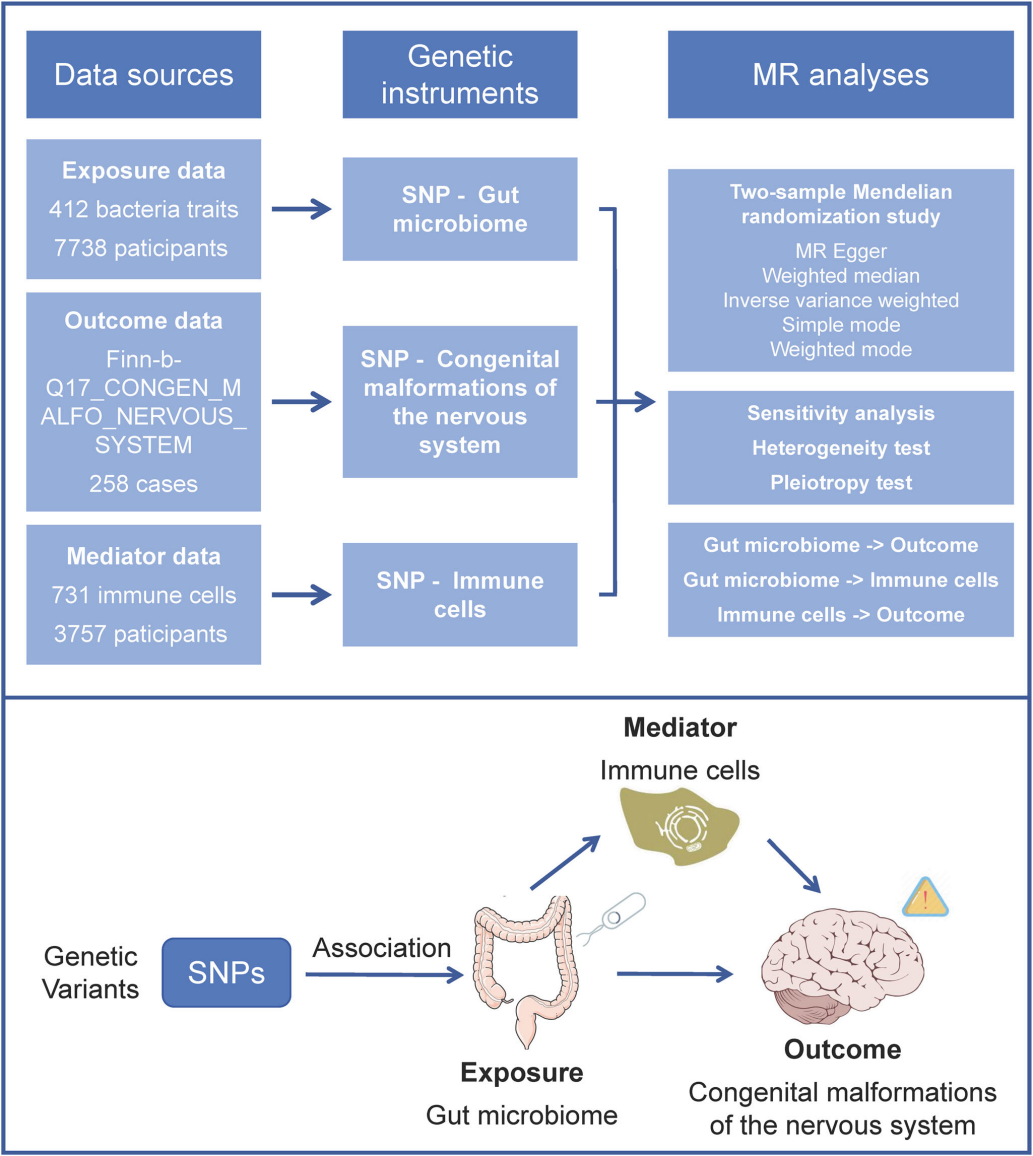
The study design using the two‐step Mendelian randomization method.

Figure 1 illustrated the study design using the TSMR method to investigate the impact of gut microbiota on congenital malformations of the nervous system and the mediating role of immune cells. The causal interpretation of MR estimates depends on three assumptions: the genetic variants used as instrumental variables, known as single nucleotide polymorphisms (SNPs), should strongly predict the exposure; they should relate to the outcome solely through the exposure; and they should be unrelated to any confounding factors of the exposure–outcome association (Wang et al. 2020). The Strengthening the Reporting of Observational Studies in Epidemiology (STROBE)‐MR checklist for our study was presented in Supporting Information Table 1 (Skrivankova et al. 2021a, 2021b).

### 2.2 Data Sources

The GWAS data for gut microbiota were from a study by Lopera‐Maya et al., involving 7738 individuals, which describes gut microbiota abundance and gut bacterial pathway abundance, totaling 412 indicators (Lopera‐Maya et al. 2022). We numbered these indicators for convenient presentation in the figures. Detailed information on these indicators and the renamed comparison table are shown in Supporting Information Table 2. Potential mediators included were immune cell markers available in genome‐wide summary statistics with adequate instrument strength and clear cellular definitions grounded in flow‐cytometry panels rather than ad hoc markers, covering those with biological plausibility for involvement in maternal–fetal immune regulation or neurodevelopment. Thus, for immune phenotype data we used the high‐resolution immune‐GWAS by Orrù et al., involving 3757 individuals, with 731 indicators (Orrù et al. 2020). The data for congenital malformations of the nervous system were from the FinnGen project, code finn‐b‐Q17_CONGEN_MALFO_NERVOUS_SYSTEM. This project is a large‐scale genomic study involving over 500,000 samples (Kurki et al. 2023). All populations included in these studies were of European descent.

### 2.3 Statistical Analysis

TSMR was used to explore the mediating role of immune cells in the impact of gut microbiota on congenital malformations of the nervous system, calculating total, mediating, and direct effects. TwoSampleMR package version 0.6.6 was used in R version 4.3.3. In the MR analysis, we applied a *p* value threshold of 1e−5 for gut microbiota and 5e−08 for immune cells to select SNPs associated with the exposures. To account for linkage disequilibrium (LD), we performed clumping using an LD Rsq threshold of 0.001 and a clumping window of 10,000 kb to prune SNPs in high LD. If an exposure SNP was unavailable in the outcome data, we used LD proxies with a minimum LD Rsq of 0.8 to substitute missing SNPs. Palindromic SNPs were allowed, and allele harmonization was applied to guarantee consistency in allele orientation across exposure and outcome data. For palindromic SNPs with a minor allele frequency greater than 0.3, realignment was performed where possible.

We utilized various methods, including Wald ratio, MR Egger, weighted median, inverse variance weighted (IVW), simple mode and weighted mode, to provide MR estimates. Additionally, a heterogeneity test to assess variability in effect estimates across SNPs and a pleiotropy test were performed to assess potential horizontal pleiotropy, ensuring the reliability of our findings.

Assuming the MR effect of gut microbiota on congenital malformations of the nervous system was *β.total*, the MR effect of gut microbiota on immune cells was *β.x*, and the MR effect of immune cells on congenital malformations of the nervous system was *β.y*, then the mediating effect was *β.x* × *β.y*, and the direct effect was *β*.total—*β.x* × *β.y*.

Our research complies with the STROBE guidelines.

## 3 Results

### 3.1 Total Causal Effects of 412 Gut Microbiota Indicators on Congenital Malformations of the Nervous System

**FIGURE 2:**
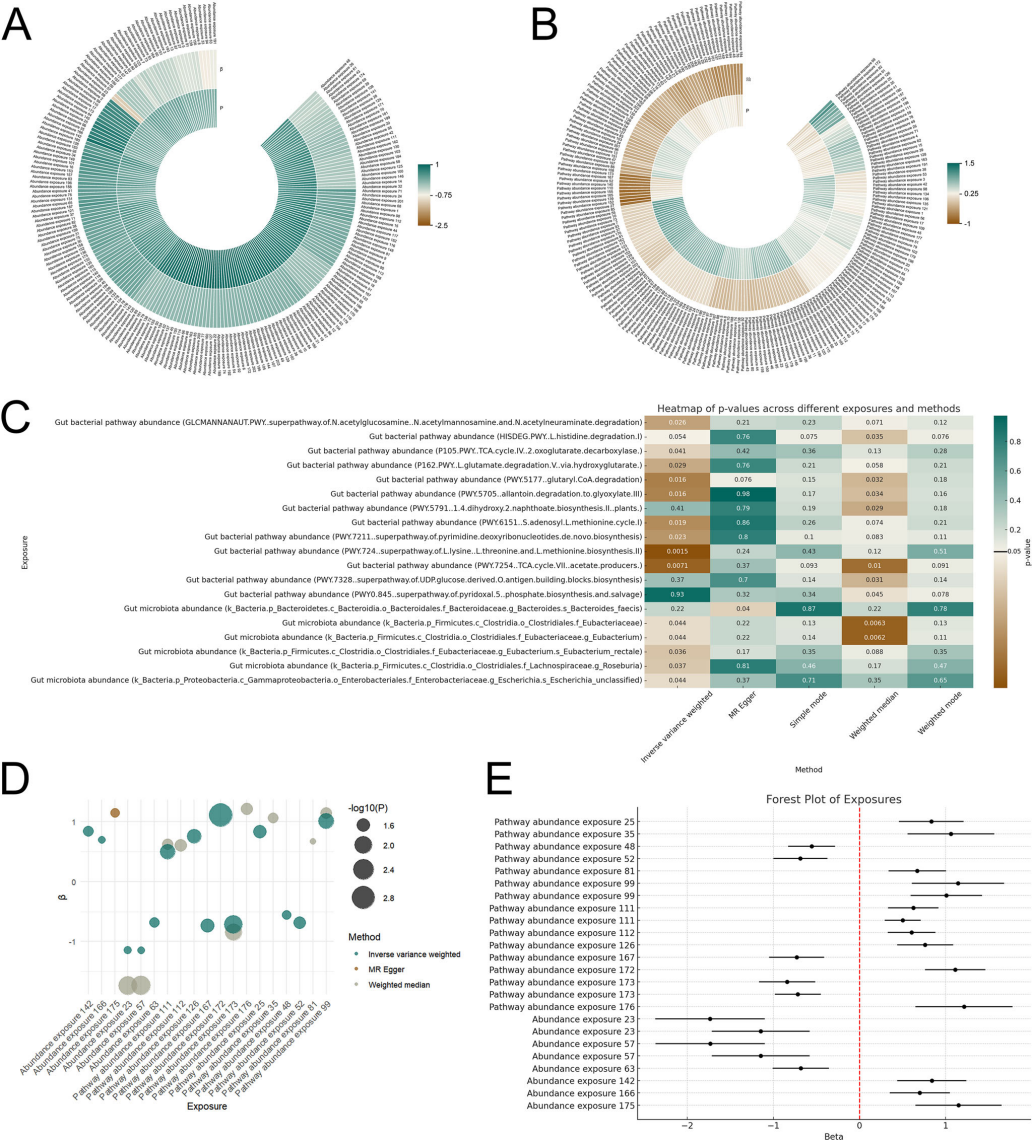
Comprehensive analysis of gut microbiota indicators on congenital malformations of the nervous system. (A) Circular heatmap of gut microbiota abundance. (B) Circular heatmap of gut bacterial pathway abundance. (C) Heatmap of the *p* of different gut microbiota indicators analyzed by five Mendelian randomization methods except Wald ratio. (D) Bubble chart showing statistically significant results. (E) Summary forest plot of statistically significant results.

Each of the 412 gut microbiota indicators was treated as exposure, and congenital malformations of the nervous system as the outcome. Results of the total causal effects using six MR methods were presented in Supporting Information Table 3. Figure 2A,B showed circular heatmaps of all effect values and statistical significance of gut microbiota abundance and gut bacterial pathway abundance from MR analysis using the IVW method. A total of 19 different gut microbiota indicators were found to have a causal association with congenital malformations of the nervous system in at least one MR method, including six on gut microbiota abundance and 13 on gut bacterial pathway abundance. Figure 2C showed the *p* of different gut microbiota indicators analyzed by five MR methods except Wald ratio. All statistically significant results were listed in Table 1, and bubble charts and summary forest plots in Figure 2D,E showed these statistically significant results.

**TABLE 1:** Mendelian randomization results with statistical significance of gut microbiota and congenital nervous system malformations risk.

Exposure	Method	*n*	*β*	SE	*p*
Gut microbiota abundance (k_Bacteria.p_Firmicutes.c_Clostridia.o_Clostridiales.f_Eubacteriaceae)	Weighted median	6	−1.735	0.636	0.006
Gut microbiota abundance (k_Bacteria.p_Firmicutes.c_Clostridia.o_Clostridiales.f_Eubacteriaceae)	Inverse variance weighted	6	−1.147	0.568	0.044
Gut microbiota abundance (k_Bacteria.p_Firmicutes.c_Clostridia.o_Clostridiales.f_Eubacteriaceae.g_Eubacterium)	Weighted median	6	−1.734	0.633	0.006
Gut microbiota abundance (k_Bacteria.p_Firmicutes.c_Clostridia.o_Clostridiales.f_Eubacteriaceae.g_Eubacterium)	Inverse variance weighted	6	−1.146	0.568	0.044
Gut microbiota abundance (k_Bacteria.p_Firmicutes.c_Clostridia.o_Clostridiales.f_Lachnospiraceae.g_Roseburia)	Inverse variance weighted	14	−0.682	0.328	0.037
Gut microbiota abundance (k_Bacteria.p_Firmicutes.c_Clostridia.o_Clostridiales.f_Eubacteriaceae.g_Eubacterium.s_Eubacterium_rectale)	Inverse variance weighted	9	0.838	0.400	0.036
Gut microbiota abundance (k_Bacteria.p_Proteobacteria.c_Gammaproteobacteria.o_Enterobacteriales.f_Enterobacteriaceae.g_Escherichia.s_Escherichia_unclassified)	Inverse variance weighted	8	0.700	0.347	0.044
Gut microbiota abundance (k_Bacteria.p_Bacteroidetes.c_Bacteroidia.o_Bacteroidales.f_Bacteroidaceae.g_Bacteroides.s_Bacteroides_faecis)	MR Egger	14	1.149	0.500	0.040
Gut bacterial pathway abundance (GLCMANNANAUT.PWY…superpathway.of.N.acetylglucosamine…N.acetylmannosamine.and.N.acetylneuraminate.degradation)	Inverse variance weighted	8	0.833	0.374	0.026
Gut bacterial pathway abundance (HISDEG.PWY…L.histidine.degradation.I)	Weighted median	11	1.060	0.504	0.035
Gut bacterial pathway abundance (P105.PWY…TCA.cycle.IV…2.oxoglutarate.decarboxylase.)	Inverse variance weighted	8	−0.556	0.272	0.041
Gut bacterial pathway abundance (P162.PWY…L.glutamate.degradation.V…via.hydroxyglutarate.)	Inverse variance weighted	11	−0.687	0.314	0.029
Gut bacterial pathway abundance (PWY0.845…superpathway.of.pyridoxal.5…phosphate.biosynthesis.and.salvage)	Weighted median	18	0.670	0.334	0.045
Gut bacterial pathway abundance (PWY.5177…glutaryl.CoA.degradation)	Weighted median	9	1.143	0.535	0.032
Gut bacterial pathway abundance (PWY.5177…glutaryl.CoA.degradation)	Inverse variance weighted	9	1.007	0.416	0.016
Gut bacterial pathway abundance (PWY.5705…allantoin.degradation.to.glyoxylate.III)	Weighted median	9	0.624	0.294	0.034
Gut bacterial pathway abundance (PWY.5705…allantoin.degradation.to.glyoxylate.III)	Inverse variance weighted	9	0.501	0.209	0.016
Gut bacterial pathway abundance (PWY.5791…1.4.dihydroxy.2.naphthoate.biosynthesis.II…plants.)	Weighted median	15	0.604	0.276	0.029
Gut bacterial pathway abundance (PWY.6151…S.adenosyl.L.methionine.cycle.I)	Inverse variance weighted	12	0.761	0.326	0.019
Gut bacterial pathway abundance (PWY.7211…superpathway.of.pyrimidine.deoxyribonucleotides.de.novo.biosynthesis)	Inverse variance weighted	13	−0.730	0.320	0.023
Gut bacterial pathway abundance (PWY.724…superpathway.of.L.lysine…L.threonine.and.L.methionine.biosynthesis.II)	Inverse variance weighted	12	1.112	0.350	0.001
Gut bacterial pathway abundance (PWY.7254…TCA.cycle.VII…acetate.producers.)	Weighted median	10	−0.841	0.328	0.010
Gut bacterial pathway abundance (PWY.7254…TCA.cycle.VII…acetate.producers.)	Inverse variance weighted	10	−0.716	0.266	0.007
Gut bacterial pathway abundance (PWY.7328…superpathway.of.UDP.glucose.derived.O.antigen.building.blocks.biosynthesis)	Weighted median	9	1.213	0.564	0.031

Abbreviations: MR, Mendelian randomization; *n*, number of SNPs; SE, standard error; SNPs, single nucleotide polymorphisms.

*p* < 0.05 was considered as statistically significant.

**FIGURE 3:**
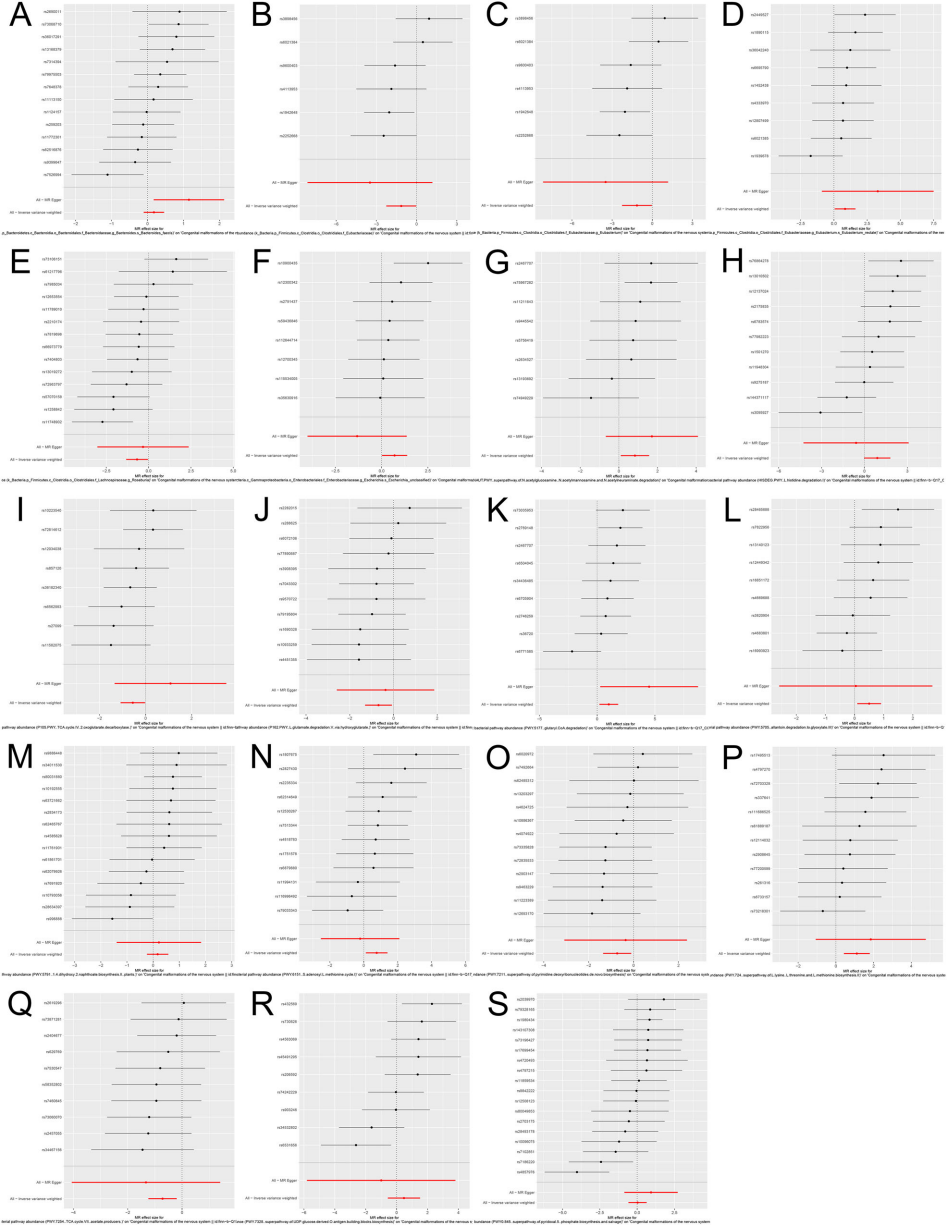
Separate forest plots demonstrated the associations of gut microbiota and their metabolic pathways with congenital malformations of the nervous system.

In evaluating the causal relationship of gut microbiota on congenital malformations of the nervous system, one family (*Eubacteriaceae*) and two genera (*Eubacterium* and *Roseburia*), both within the order *Clostridiales*, showed a negative correlation with congenital malformations of the nervous system. Three species, however, showed a positive correlation (*Eubacterium rectale*, *Escherichia unclassified*, and *Bacteroides faecis*). Among these, the family *Eubacteriaceae* had the strongest impact on the risk of congenital malformations of the nervous system (*β* = −1.735, SE = 0.636, *p* = 0.006; Figure 3A–F.

In assessing the causal relationship of gut bacterial pathway abundance with congenital malformations of the nervous system, several pathways showed a positive correlation with congenital malformations, such as GLCMANNANAUT.PWY (N‐acetylglucosamine, N‐acetylmannosamine, and N‐acetylneuraminic acid degradation superpathway), HISDEG.PWY (L‐histidine degradation pathway I), PWY0.845 (pyridoxal 5′‐phosphate synthesis and salvage superpathway), and several others. Conversely, pathways such as P105.PWY (tricarboxylic acid cycle IV), P162.PWY (L‐glutamate degradation pathway V), and PWY.724 (superpathway of lysine, threonine, and methionine biosynthesis II) showed a negative correlation with the risk of congenital malformations of the nervous system (Figure 3G–S).

We used funnel plots to detect heterogeneity among genetic variants. As shown in Supporting Information Figure 1, the funnel plots appeared symmetrical, indicating no significant heterogeneity. Cochran's *Q*‐test was used to assess heterogeneity across SNPs (Supporting Information Table 4). All the *p* were greater than 0.05, indicating no significant heterogeneity, which supports the robustness of our MR analysis. Horizontal pleiotropy refers to SNPs affecting outcomes through pathways other than exposure, which can bias causal estimates in MR analysis. To evaluate the presence of horizontal pleiotropy, we used the MR‐Egger regression method. The MR‐Egger intercept provided an estimate of horizontal pleiotropy magnitude. In our analysis, all MR‐Egger intercepts were close to zero with *p* greater than 0.05, suggesting no significant horizontal pleiotropy affecting the results (Supporting Information Table 5). This indicated that the SNPs used in our MR analysis did not exhibit pleiotropy that would bias causal estimates, thereby supporting the validity of our study findings.

### 3.2 Identification of Nine Immune Cell Indicators as Positive Mediators in the Influence of Gut Microbiota on Congenital Malformations of the Nervous System

**FIGURE 4:**
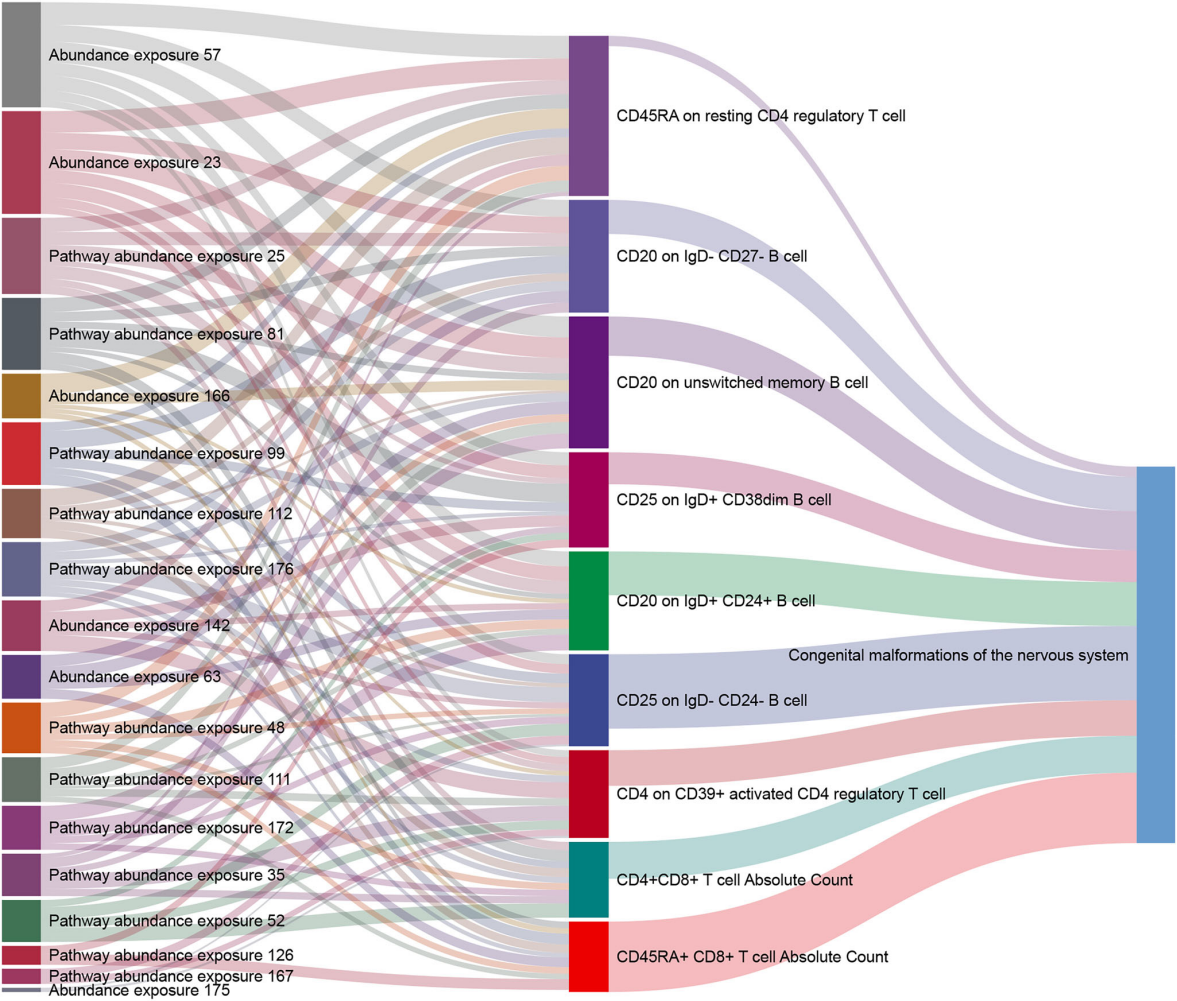
The interactive Sankey diagram illustrated immune cells as mediators.

In TSMR using the IVW method, nine immune cells were identified as mediators in the influence of gut microbiota on congenital malformations of the nervous system. As shown in Figure 4, the interactive Sankey diagram illustrated immune cells as mediators in the impact of gut microbiota on congenital malformations of the nervous system.

Among the 19 gut microbiota taxa associated with congenital malformations of the nervous system, 18 were significantly associated with at least one of the nine immune cell markers (Table 2). We found that different taxa in the phyla *Firmicutes* and *Proteobacteria*, such as the family *Eubacteriaceae* and genera *Eubacterium* and *Roseburia*, significantly affected marker expression on B cells and T cells. For example, CD20 expression in IgD^+^ CD24^+^ and IgD^−^ CD27^−^ B cells was significantly associated with gut taxa like the family *Lachnospiraceae*. Additionally, metabolic pathways, such as N‐acetylglucosamine degradation, tricarboxylic acid cycle, and L‐histidine degradation, showed complex positive or negative associations with marker expression in various immune cell types (*p* < 0.05). These pathways impacted CD4^+^CD8^+^ T cell absolute counts and CD45RA expression in resting CD4 regulatory T cells, indicating an important role of gut microbiota and its metabolites in modulating immune response.

**TABLE 2:** Significant immune cells as potential mediators and their association with gut microbiota.

Mediator	Exposure	*β*	SE	*p*
CD20 on IgD+ CD24+ B cell	Gut microbiota abundance (k_Bacteria.p_Firmicutes.c_Clostridia.o_Clostridiales.f_Eubacteriaceae)	−0.258	0.051	< 0.001
	Gut microbiota abundance (k_Bacteria.p_Firmicutes.c_Clostridia.o_Clostridiales.f_Eubacteriaceae.g_Eubacterium)	−0.267	0.051	< 0.001
	Gut microbiota abundance (k_Bacteria.p_Firmicutes.c_Clostridia.o_Clostridiales.f_Lachnospiraceae.g_Roseburia)	−0.176	0.042	< 0.001
	Gut microbiota abundance (k_Bacteria.p_Firmicutes.c_Clostridia.o_Clostridiales.f_Eubacteriaceae.g_Eubacterium.s_Eubacterium_rectale)	−0.113	0.043	0.009
	Gut microbiota abundance (k_Bacteria.p_Proteobacteria.c_Gammaproteobacteria.o_Enterobacteriales.f_Enterobacteriaceae.g_Escherichia.s_Escherichia_unclassified)	−0.075	0.027	0.005
	Gut bacterial pathway abundance (GLCMANNANAUT.PWY…superpathway.of.N.acetylglucosamine…N.acetylmannosamine.and.N.acetylneuraminate.degradation)	−0.232	0.048	< 0.001
	Gut bacterial pathway abundance (P105.PWY…TCA.cycle.IV…2.oxoglutarate.decarboxylase.)	0.166	0.031	< 0.001
	Gut bacterial pathway abundance (PWY0.845…superpathway.of.pyridoxal.5…phosphate.biosynthesis.and.salvage)	−0.090	0.037	0.015
	Gut bacterial pathway abundance (PWY.5705…allantoin.degradation.to.glyoxylate.III)	−0.105	0.014	< 0.001
	Gut bacterial pathway abundance (PWY.724…superpathway.of.L.lysine…L.threonine.and.L.methionine.biosynthesis.II)	−0.281	0.045	< 0.001
CD20 on IgD− CD27− B cell	Gut microbiota abundance (k_Bacteria.p_Firmicutes.c_Clostridia.o_Clostridiales.f_Eubacteriaceae)	−0.293	0.063	< 0.001
	Gut microbiota abundance (k_Bacteria.p_Firmicutes.c_Clostridia.o_Clostridiales.f_Eubacteriaceae.g_Eubacterium)	−0.300	0.064	< 0.001
	Gut microbiota abundance (k_Bacteria.p_Firmicutes.c_Clostridia.o_Clostridiales.f_Lachnospiraceae.g_Roseburia)	0.210	0.053	< 0.001
	Gut bacterial pathway abundance (GLCMANNANAUT.PWY…superpathway.of.N.acetylglucosamine…N.acetylmannosamine.and.N.acetylneuraminate.degradation)	−0.232	0.056	< 0.001
	Gut bacterial pathway abundance (HISDEG.PWY…L.histidine.degradation.I)	−0.179	0.035	< 0.001
	Gut bacterial pathway abundance (PWY0.845…superpathway.of.pyridoxal.5…phosphate.biosynthesis.and.salvage)	−0.171	0.038	< 0.001
	Gut bacterial pathway abundance (PWY.5177…glutaryl.CoA.degradation)	−0.312	0.068	< 0.001
	Gut bacterial pathway abundance (PWY.5791…1.4.dihydroxy.2.naphthoate.biosynthesis.II…plants.)	−0.140	0.025	< 0.001
	Gut bacterial pathway abundance (PWY.7328…superpathway.of.UDP.glucose.derived.O.antigen.building.blocks.biosynthesis)	0.172	0.051	< 0.001
CD20 on unswitched memory B cell	Gut microbiota abundance (k_Bacteria.p_Firmicutes.c_Clostridia.o_Clostridiales.f_Eubacteriaceae)	−0.366	0.051	< 0.001
	Gut microbiota abundance (k_Bacteria.p_Firmicutes.c_Clostridia.o_Clostridiales.f_Eubacteriaceae.g_Eubacterium)	−0.373	0.051	< 0.001
	Gut microbiota abundance (k_Bacteria.p_Firmicutes.c_Clostridia.o_Clostridiales.f_Lachnospiraceae.g_Roseburia)	−0.221	0.051	< 0.001
	Gut microbiota abundance (k_Bacteria.p_Proteobacteria.c_Gammaproteobacteria.o_Enterobacteriales.f_Enterobacteriaceae.g_Escherichia.s_Escherichia_unclassified)	−0.185	0.027	< 0.001
	Gut bacterial pathway abundance (GLCMANNANAUT.PWY…superpathway.of.N.acetylglucosamine…N.acetylmannosamine.and.N.acetylneuraminate.degradation)	−0.273	0.055	< 0.001
	Gut bacterial pathway abundance (P105.PWY…TCA.cycle.IV…2.oxoglutarate.decarboxylase.)	0.140	0.032	< 0.001
	Gut bacterial pathway abundance (PWY0.845…superpathway.of.pyridoxal.5…phosphate.biosynthesis.and.salvage)	−0.121	0.037	0.001
	Gut bacterial pathway abundance (PWY.5705…allantoin.degradation.to.glyoxylate.III)	−0.207	0.016	< 0.001
	Gut bacterial pathway abundance (PWY.5791…1.4.dihydroxy.2.naphthoate.biosynthesis.II…plants.)	0.047	0.022	0.037
	Gut bacterial pathway abundance (PWY.724…superpathway.of.L.lysine…L.threonine.and.L.methionine.biosynthesis.II)	−0.265	0.045	< 0.001
	Gut bacterial pathway abundance (PWY.7328…superpathway.of.UDP.glucose.derived.O.antigen.building.blocks.biosynthesis)	−0.153	0.037	< 0.001
CD25 on IgD+ CD38dim B cell	Gut microbiota abundance (k_Bacteria.p_Firmicutes.c_Clostridia.o_Clostridiales.f_Eubacteriaceae)	−0.232	0.052	< 0.001
	Gut microbiota abundance (k_Bacteria.p_Firmicutes.c_Clostridia.o_Clostridiales.f_Eubacteriaceae.g_Eubacterium)	−0.233	0.052	< 0.001
	Gut microbiota abundance (k_Bacteria.p_Firmicutes.c_Clostridia.o_Clostridiales.f_Eubacteriaceae.g_Eubacterium.s_Eubacterium_rectale)	0.199	0.047	< 0.001
	Gut bacterial pathway abundance (GLCMANNANAUT.PWY…superpathway.of.N.acetylglucosamine…N.acetylmannosamine.and.N.acetylneuraminate.degradation)	0.096	0.045	0.033
	Gut bacterial pathway abundance (HISDEG.PWY…L.histidine.degradation.I)	−0.109	0.034	0.002
	Gut bacterial pathway abundance (P162.PWY…L.glutamate.degradation.V…via.hydroxyglutarate.)	0.122	0.054	0.023
	Gut bacterial pathway abundance (PWY0.845…superpathway.of.pyridoxal.5…phosphate.biosynthesis.and.salvage)	−0.331	0.053	< 0.001
	Gut bacterial pathway abundance (PWY.5177…glutaryl.CoA.degradation)	0.164	0.054	0.002
	Gut bacterial pathway abundance (PWY.6151…S.adenosyl.L.methionine.cycle.I)	−0.144	0.064	0.025
	Gut bacterial pathway abundance (PWY.7328…superpathway.of.UDP.glucose.derived.O.antigen.building.blocks.biosynthesis)	−0.068	0.032	0.035
CD25 on IgD− CD24− B cell	Gut microbiota abundance (k_Bacteria.p_Firmicutes.c_Clostridia.o_Clostridiales.f_Eubacteriaceae)	−0.169	0.051	< 0.001
	Gut microbiota abundance (k_Bacteria.p_Firmicutes.c_Clostridia.o_Clostridiales.f_Eubacteriaceae.g_Eubacterium)	−0.175	0.052	< 0.001
	Gut microbiota abundance (k_Bacteria.p_Firmicutes.c_Clostridia.o_Clostridiales.f_Eubacteriaceae.g_Eubacterium.s_Eubacterium_rectale)	0.108	0.044	0.013
	Gut microbiota abundance (k_Bacteria.p_Bacteroidetes.c_Bacteroidia.o_Bacteroidales.f_Bacteroidaceae.g_Bacteroides.s_Bacteroides_faecis)	0.034	0.017	0.047
	Gut bacterial pathway abundance (P105.PWY…TCA.cycle.IV…2.oxoglutarate.decarboxylase.)	0.094	0.030	0.002
	Gut bacterial pathway abundance (P162.PWY…L.glutamate.degradation.V…via.hydroxyglutarate.)	0.213	0.053	< 0.001
	Gut bacterial pathway abundance (PWY.5177…glutaryl.CoA.degradation)	−0.174	0.054	0.001
	Gut bacterial pathway abundance (PWY.5705…allantoin.degradation.to.glyoxylate.III)	−0.052	0.013	< 0.001
	Gut bacterial pathway abundance (PWY.5791…1.4.dihydroxy.2.naphthoate.biosynthesis.II…plants.)	−0.069	0.023	0.002
	Gut bacterial pathway abundance (PWY.7211…superpathway.of.pyrimidine.deoxyribonucleotides.de.novo.biosynthesis)	0.156	0.053	0.003
	Gut bacterial pathway abundance (PWY.724…superpathway.of.L.lysine…L.threonine.and.L.methionine.biosynthesis.II)	0.124	0.063	0.050
	Gut bacterial pathway abundance (PWY.7328…superpathway.of.UDP.glucose.derived.O.antigen.building.blocks.biosynthesis)	−0.270	0.033	< 0.001
CD4 on CD39+ activated CD4 regulatory T cell	Gut microbiota abundance (k_Bacteria.p_Firmicutes.c_Clostridia.o_Clostridiales.f_Eubacteriaceae)	0.126	0.056	0.024
	Gut microbiota abundance (k_Bacteria.p_Firmicutes.c_Clostridia.o_Clostridiales.f_Eubacteriaceae.g_Eubacterium)	0.122	0.057	0.031
	Gut microbiota abundance (k_Bacteria.p_Firmicutes.c_Clostridia.o_Clostridiales.f_Eubacteriaceae.g_Eubacterium.s_Eubacterium_rectale)	0.282	0.053	< 0.001
	Gut microbiota abundance (k_Bacteria.p_Proteobacteria.c_Gammaproteobacteria.o_Enterobacteriales.f_Enterobacteriaceae.g_Escherichia.s_Escherichia_unclassified)	0.078	0.029	0.008
	Gut microbiota abundance (k_Bacteria.p_Bacteroidetes.c_Bacteroidia.o_Bacteroidales.f_Bacteroidaceae.g_Bacteroides.s_Bacteroides_faecis)	0.040	0.020	0.045
	Gut bacterial pathway abundance (GLCMANNANAUT.PWY…superpathway.of.N.acetylglucosamine…N.acetylmannosamine.and.N.acetylneuraminate.degradation)	0.125	0.049	0.011
	Gut bacterial pathway abundance (HISDEG.PWY…L.histidine.degradation.I)	0.263	0.039	< 0.001
	Gut bacterial pathway abundance (P162.PWY…L.glutamate.degradation.V…via.hydroxyglutarate.)	0.158	0.072	0.027
	Gut bacterial pathway abundance (PWY.5705…allantoin.degradation.to.glyoxylate.III)	0.134	0.014	< 0.001
	Gut bacterial pathway abundance (PWY.7211…superpathway.of.pyrimidine.deoxyribonucleotides.de.novo.biosynthesis)	0.114	0.058	0.049
	Gut bacterial pathway abundance (PWY.7328…superpathway.of.UDP.glucose.derived.O.antigen.building.blocks.biosynthesis)	0.121	0.035	< 0.001
CD4+CD8+ T cell Absolute Count	Gut bacterial pathway abundance (GLCMANNANAUT.PWY…superpathway.of.N.acetylglucosamine…N.acetylmannosamine.and.N.acetylneuraminate.degradation)	0.147	0.050	0.003
	Gut bacterial pathway abundance (HISDEG.PWY…L.histidine.degradation.I)	0.129	0.034	< 0.001
	Gut bacterial pathway abundance (P105.PWY…TCA.cycle.IV…2.oxoglutarate.decarboxylase.)	0.122	0.044	0.006
	Gut bacterial pathway abundance (P162.PWY…L.glutamate.degradation.V…via.hydroxyglutarate.)	−0.255	0.058	< 0.001
	Gut bacterial pathway abundance (PWY0.845…superpathway.of.pyridoxal.5…phosphate.biosynthesis.and.salvage)	−0.202	0.037	< 0.001
	Gut bacterial pathway abundance (PWY.5177…glutaryl.CoA.degradation)	−0.120	0.053	0.023
	Gut bacterial pathway abundance (PWY.5791…1.4.dihydroxy.2.naphthoate.biosynthesis.II…plants.)	0.158	0.022	< 0.001
	Gut bacterial pathway abundance (PWY.724…superpathway.of.L.lysine…L.threonine.and.L.methionine.biosynthesis.II)	−0.112	0.045	0.013
	Gut bacterial pathway abundance (PWY.7328…superpathway.of.UDP.glucose.derived.O.antigen.building.blocks.biosynthesis)	−0.106	0.032	< 0.001
CD45RA on resting CD4 regulatory T cell	Gut microbiota abundance (k_Bacteria.p_Firmicutes.c_Clostridia.o_Clostridiales.f_Eubacteriaceae)	−0.385	0.053	< 0.001
	Gut microbiota abundance (k_Bacteria.p_Firmicutes.c_Clostridia.o_Clostridiales.f_Eubacteriaceae.g_Eubacterium)	−0.404	0.054	< 0.001
	Gut microbiota abundance (k_Bacteria.p_Firmicutes.c_Clostridia.o_Clostridiales.f_Eubacteriaceae.g_Eubacterium.s_Eubacterium_rectale)	−0.200	0.046	< 0.001
	Gut microbiota abundance (k_Bacteria.p_Proteobacteria.c_Gammaproteobacteria.o_Enterobacteriales.f_Enterobacteriaceae.g_Escherichia.s_Escherichia_unclassified)	0.358	0.028	< 0.001
	Gut bacterial pathway abundance (GLCMANNANAUT.PWY…superpathway.of.N.acetylglucosamine…N.acetylmannosamine.and.N.acetylneuraminate.degradation)	−0.253	0.055	< 0.001
	Gut bacterial pathway abundance (HISDEG.PWY…L.histidine.degradation.I)	0.075	0.036	0.037
	Gut bacterial pathway abundance (P105.PWY…TCA.cycle.IV…2.oxoglutarate.decarboxylase.)	−0.262	0.033	< 0.001
	Gut bacterial pathway abundance (PWY0.845…superpathway.of.pyridoxal.5…phosphate.biosynthesis.and.salvage)	0.250	0.055	< 0.001
	Gut bacterial pathway abundance (PWY.5177…glutaryl.CoA.degradation)	0.155	0.056	0.005
	Gut bacterial pathway abundance (PWY.5705…allantoin.degradation.to.glyoxylate.III)	−0.202	0.014	< 0.001
	Gut bacterial pathway abundance (PWY.5791…1.4.dihydroxy.2.naphthoate.biosynthesis.II…plants.)	−0.310	0.028	< 0.001
CD45RA+ CD8+ T cell Absolute Count	Gut microbiota abundance (k_Bacteria.p_Firmicutes.c_Clostridia.o_Clostridiales.f_Lachnospiraceae.g_Roseburia)	−0.173	0.050	< 0.001
	Gut microbiota abundance (k_Bacteria.p_Proteobacteria.c_Gammaproteobacteria.o_Enterobacteriales.f_Enterobacteriaceae.g_Escherichia.s_Escherichia_unclassified)	0.098	0.024	< 0.001
	Gut bacterial pathway abundance (P105.PWY…TCA.cycle.IV…2.oxoglutarate.decarboxylase.)	0.118	0.026	< 0.001
	Gut bacterial pathway abundance (PWY0.845…superpathway.of.pyridoxal.5…phosphate.biosynthesis.and.salvage)	0.116	0.033	< 0.001
	Gut bacterial pathway abundance (PWY.5177…glutaryl.CoA.degradation)	0.191	0.052	< 0.001
	Gut bacterial pathway abundance (PWY.5705…allantoin.degradation.to.glyoxylate.III)	0.096	0.012	< 0.001
	Gut bacterial pathway abundance (PWY.5791…1.4.dihydroxy.2.naphthoate.biosynthesis.II…plants.)	0.154	0.020	< 0.001
	Gut bacterial pathway abundance (PWY.6151…S.adenosyl.L.methionine.cycle.I)	0.192	0.052	< 0.001
	Gut bacterial pathway abundance (PWY.7328…superpathway.of.UDP.glucose.derived.O.antigen.building.blocks.biosynthesis)	−0.079	0.028	0.005

Abbreviation: SE, standard error.

*p* < 0.05 was considered as statistically significant.

The causal relationships of the nine immune cell indicators with congenital malformations of the nervous system were presented in Table 3. For example, CD45RA^+^ CD8^+^ T cell absolute count, CD20 on IgD^+^ CD24^+^ B cell, CD20 on IgD^−^ CD27^−^ B cell, and CD20 on unswitched memory B cell (*β* values of −0.781, −0.607, −0.701, respectively, *p* < 0.05) showed negative correlations, suggesting that increased levels of these immune cells may reduce nervous system abnormalities. Conversely, CD4^+^CD8^+^ T cell absolute count, CD25 on IgD^+^ CD38dim B cell, and CD25 on IgD^−^ CD24^−^ B cell (*β* values of 0.657, 0.569, and 1.325, respectively, *p* < 0.05) were positively associated with higher risk of congenital malformations of the nervous system.

**TABLE 3:** Significant immune cells as potential mediators and their association with congenital nervous system malformations.

Mediator	*n*	*Β*	SE	*p*	OR
CD45RA+ CD8+ T cell Absolute Count	2	−1.255	0.504	0.013	0.285
CD4+CD8+ T cell Absolute Count	2	0.657	0.320	0.040	1.930
CD20 on IgD+ CD24+ B cell	2	−0.781	0.372	0.036	0.458
CD20 on IgD− CD27− B cell	4	−0.607	0.302	0.045	0.545
CD20 on unswitched memory B cell	4	−0.701	0.311	0.024	0.496
CD25 on IgD+ CD38dim B cell	3	0.569	0.258	0.028	1.767
CD25 on IgD− CD24− B cell	2	1.325	0.507	0.009	3.761
CD4 on CD39+ activated CD4 regulatory T cell	3	0.635	0.279	0.023	1.887
CD45RA on resting CD4 regulatory T cell	5	−0.183	0.090	0.041	0.833

Abbreviations: *n*, number of SNPs; OR, odds ratio; SE, standard error; SNPs, single nucleotide polymorphisms.

*p* < 0.05 was considered as statistically significant.

Table 4 summarized the mediating role of immune cells in the influence of gut microbiota on congenital malformations of the nervous system, providing total, mediating, and direct effects. For example, the gut bacterial pathway GLCMANNANAUT.PWY (N‐acetylglucosamine, N‐acetylmannosamine, and N‐acetylneuraminic acid degradation superpathway) showed a positive association with CD4^+^CD8^+^ T cell absolute count (*β.x* = 0.147, *p.x* = 0.003, OR*.x* = 1.158), with a direct positive effect on congenital malformations of the nervous system (*β.y* = 0.657, *p.y* = 0.040, OR*.y* = 1.930) and a mediating effect of 0.097. Conversely, CD20 in IgD^+^ CD24^+^ B cells showed a negative mediating effect (*β.x* = −0.232, *p.x* < 0.001, OR*.x* = 0.793) with a direct negative effect on congenital malformations of the nervous system (*β.y* = −0.781, *p.y* = 0.036, OR*.y* = 0.458), with a mediating effect of 0.181. These results indicated that different immune cell types, with their marker expressions and counts, exhibited complex associations between gut microbiota‐related indicators and congenital malformations of the nervous system.

**TABLE 4:** Nine immune cells as mediators in gut microbiota's effect on congenital nervous system malformations.

Exposure	Mediator	*β.x*	SE*.x*	*p.x*	OR*.x*	*β.y*	SE*.y*	*p.y*	OR*.y*	Mediating effect	Total effect	Direct effect
Gut microbiota abundance (k_Bacteria.p_Firmicutes.c_Clostridia.o_Clostridiales.f_Eubacteriaceae)	CD20 on IgD+ CD24+ B cell	−0.258	0.051	< 0.001	0.773	−0.781	0.372	0.036	0.458	0.201	−1.147	−1.348
	CD20 on IgD− CD27− B cell	−0.293	0.063	< 0.001	0.746	−0.607	0.302	0.045	0.545	0.178	−1.147	−1.325
	CD20 on unswitched memory B cell	−0.366	0.051	< 0.001	0.693	−0.701	0.311	0.024	0.496	0.256	−1.147	−1.403
	CD25 on IgD+ CD38dim B cell	−0.232	0.052	< 0.001	0.793	0.569	0.258	0.028	1.767	−0.132	−1.147	−1.014
	CD25 on IgD− CD24‐ B cell	−0.169	0.051	0.001	0.844	1.325	0.507	0.009	3.761	−0.224	−1.147	−0.922
	CD4 on CD39+ activated CD4 regulatory T cell	0.126	0.056	0.024	1.135	0.635	0.279	0.023	1.887	0.080	−1.147	−1.227
	CD45RA on resting CD4 regulatory T cell	−0.385	0.053	< 0.001	0.680	−0.183	0.090	0.041	0.833	0.070	−1.147	−1.217
Gut microbiota abundance (k_Bacteria.p_Firmicutes.c_Clostridia.o_Clostridiales.f_Eubacteriaceae.g_Eubacterium)	CD20 on IgD+ CD24+ B cell	−0.267	0.051	< 0.001	0.766	−0.781	0.372	0.036	0.458	0.208	−1.146	−1.355
	CD20 on IgD− CD27− B cell	−0.300	0.064	< 0.001	0.741	−0.607	0.302	0.045	0.545	0.182	−1.146	−1.328
	CD20 on unswitched memory B cell	−0.373	0.051	< 0.001	0.689	−0.701	0.311	0.024	0.496	0.261	−1.146	−1.408
	CD25 on IgD+ CD38dim B cell	−0.233	0.052	< 0.001	0.792	0.569	0.258	0.028	1.767	−0.132	−1.146	−1.014
	CD25 on IgD− CD24‐ B cell	−0.175	0.052	0.001	0.840	1.325	0.507	0.009	3.761	−0.231	−1.146	−0.915
	CD4 on CD39+ activated CD4 regulatory T cell	0.122	0.057	0.031	1.130	0.635	0.279	0.023	1.887	0.078	−1.146	−1.224
	CD45RA on resting CD4 regulatory T cell	−0.404	0.054	< 0.001	0.668	−0.183	0.090	0.041	0.833	0.074	−1.146	−1.220
Gut microbiota abundance (k_Bacteria.p_Firmicutes.c_Clostridia.o_Clostridiales.f_Lachnospiraceae.g_Roseburia)	CD45RA+ CD8+ T cell Absolute Count	−0.173	0.050	0.001	0.841	−1.255	0.504	0.013	0.285	0.217	−0.682	−0.899
	CD20 on IgD+ CD24+ B cell	−0.176	0.042	< 0.001	0.838	−0.781	0.372	0.036	0.458	0.138	−0.682	−0.820
	CD20 on IgD− CD27− B cell	0.210	0.053	< 0.001	1.233	−0.607	0.302	0.045	0.545	−0.127	−0.682	−0.555
	CD20 on unswitched memory B cell	−0.221	0.051	< 0.001	0.802	−0.701	0.311	0.024	0.496	0.155	−0.682	−0.837
Gut microbiota abundance (k_Bacteria.p_Firmicutes.c_Clostridia.o_Clostridiales.f_Eubacteriaceae.g_Eubacterium.s_Eubacterium_rectale)	CD20 on IgD+ CD24+ B cell	−0.113	0.043	0.009	0.893	−0.781	0.372	0.036	0.458	0.088	0.838	0.750
	CD25 on IgD+ CD38dim B cell	0.199	0.047	< 0.001	1.220	0.569	0.258	0.028	1.767	0.113	0.838	0.725
	CD25 on IgD− CD24− B cell	0.108	0.044	0.013	1.114	1.325	0.507	0.009	3.761	0.143	0.838	0.695
	CD4 on CD39+ activated CD4 regulatory T cell	0.282	0.053	< 0.001	1.326	0.635	0.279	0.023	1.887	0.179	0.838	0.659
	CD45RA on resting CD4 regulatory T cell	−0.200	0.046	< 0.001	0.819	−0.183	0.090	0.041	0.833	0.036	0.838	0.802
Gut microbiota abundance (k_Bacteria.p_Proteobacteria.c_Gammaproteobacteria.o_Enterobacteriales.f_Enterobacteriaceae.g_Escherichia.s_Escherichia_unclassified)	CD45RA+ CD8+ T cell Absolute Count	0.098	0.024	< 0.001	1.103	−1.255	0.504	0.013	0.285	−0.124	0.700	0.823
	CD20 on IgD+ CD24+ B cell	−0.075	0.027	0.005	0.928	−0.781	0.372	0.036	0.458	0.059	0.700	0.641
	CD20 on unswitched memory B cell	−0.185	0.027	< 0.001	0.831	−0.701	0.311	0.024	0.496	0.130	0.700	0.570
	CD4 on CD39+ activated CD4 regulatory T cell	0.078	0.029	0.008	1.081	0.635	0.279	0.023	1.887	0.049	0.700	0.651
	CD45RA on resting CD4 regulatory T cell	0.358	0.028	< 0.001	1.430	−0.183	0.090	0.041	0.833	−0.065	0.700	0.765
Gut microbiota abundance (k_Bacteria.p_Bacteroidetes.c_Bacteroidia.o_Bacteroidales.f_Bacteroidaceae.g_Bacteroides.s_Bacteroides_faecis)	CD25 on IgD− CD24‐ B cell	0.034	0.017	0.047	1.035	1.325	0.507	0.009	3.761	0.045	0.176	0.131
	CD4 on CD39+ activated CD4 regulatory T cell	0.040	0.020	0.045	1.041	0.635	0.279	0.023	1.887	0.025	0.176	0.151
Gut bacterial pathway abundance (GLCMANNANAUT.PWY…superpathway.of.N.acetylglucosamine…N.acetylmannosamine.and.N.acetylneuraminate.degradation)	CD4+CD8+ T cell Absolute Count	0.147	0.050	0.003	1.158	0.657	0.320	0.040	1.930	0.097	0.833	0.736
	CD20 on IgD+ CD24+ B cell	−0.232	0.048	< 0.001	0.793	−0.781	0.372	0.036	0.458	0.181	0.833	0.652
	CD20 on IgD− CD27− B cell	−0.232	0.056	< 0.001	0.793	−0.607	0.302	0.045	0.545	0.141	0.833	0.692
	CD20 on unswitched memory B cell	−0.273	0.055	< 0.001	0.761	−0.701	0.311	0.024	0.496	0.191	0.833	0.642
	CD25 on IgD+ CD38dim B cell	0.096	0.045	0.033	1.100	0.569	0.258	0.028	1.767	0.054	0.833	0.778
	CD4 on CD39+ activated CD4 regulatory T cell	0.125	0.049	0.011	1.133	0.635	0.279	0.023	1.887	0.079	0.833	0.754
	CD45RA on resting CD4 regulatory T cell	−0.253	0.055	< 0.001	0.776	−0.183	0.090	0.041	0.833	0.046	0.833	0.787
Gut bacterial pathway abundance (HISDEG.PWY…L.histidine.degradation.I)	CD4+CD8+ T cell Absolute Count	0.129	0.034	< 0.001	1.137	0.657	0.320	0.040	1.930	0.085	0.897	0.812
	CD20 on IgD− CD27− B cell	−0.179	0.035	< 0.001	0.836	−0.607	0.302	0.045	0.545	0.109	0.897	0.788
	CD25 on IgD+ CD38dim B cell	−0.109	0.034	0.002	0.897	0.569	0.258	0.028	1.767	−0.062	0.897	0.959
	CD4 on CD39+ activated CD4 regulatory T cell	0.263	0.039	< 0.001	1.301	0.635	0.279	0.023	1.887	0.167	0.897	0.729
	CD45RA on resting CD4 regulatory T cell	0.075	0.036	0.037	1.078	−0.183	0.090	0.041	0.833	−0.014	0.897	0.910
Gut bacterial pathway abundance (P105.PWY…TCA.cycle.IV…2.oxoglutarate.decarboxylase.)	CD45RA+ CD8+ T cell Absolute Count	0.118	0.026	< 0.001	1.126	−1.255	0.504	0.013	0.285	−0.149	−0.556	−0.408
	CD4+CD8+ T cell Absolute Count	0.122	0.044	0.006	1.130	0.657	0.320	0.040	1.930	0.080	−0.556	−0.637
	CD20 on IgD+ CD24+ B cell	0.166	0.031	< 0.001	1.181	−0.781	0.372	0.036	0.458	−0.130	−0.556	−0.426
	CD20 on unswitched memory B cell	0.140	0.032	< 0.001	1.150	−0.701	0.311	0.024	0.496	−0.098	−0.556	−0.458
	CD25 on IgD− CD24− B cell	0.094	0.030	0.002	1.098	1.325	0.507	0.009	3.761	0.124	−0.556	−0.680
	CD45RA on resting CD4 regulatory T cell	−0.262	0.033	< 0.001	0.770	−0.183	0.090	0.041	0.833	0.048	−0.556	−0.604
Gut bacterial pathway abundance (P162.PWY…L.glutamate.degradation.V…via.hydroxyglutarate.)	CD4+CD8+ T cell Absolute Count	−0.255	0.058	< 0.001	0.775	0.657	0.320	0.040	1.930	−0.168	−0.687	−0.519
	CD25 on IgD+ CD38dim B cell	0.122	0.054	0.023	1.129	0.569	0.258	0.028	1.767	0.069	−0.687	−0.756
	CD25 on IgD− CD24− B cell	0.213	0.053	< 0.001	1.238	1.325	0.507	0.009	3.761	0.283	−0.687	−0.970
	CD4 on CD39+ activated CD4 regulatory T cell	0.158	0.072	0.027	1.171	0.635	0.279	0.023	1.887	0.100	−0.687	−0.787
Gut bacterial pathway abundance (PWY0.845…superpathway.of.pyridoxal.5…phosphate.biosynthesis.and.salvage)	CD45RA+ CD8+ T cell Absolute Count	0.116	0.033	< 0.001	1.123	−1.255	0.504	0.013	0.285	−0.145	0.027	0.172
	CD4+CD8+ T cell Absolute Count	−0.202	0.037	< 0.001	0.817	0.657	0.320	0.040	1.930	−0.133	0.027	0.160
	CD20 on IgD+ CD24+ B cell	−0.090	0.037	0.015	0.914	−0.781	0.372	0.036	0.458	0.070	0.027	−0.043
	CD20 on IgD− CD27− B cell	−0.171	0.038	< 0.001	0.843	−0.607	0.302	0.045	0.545	0.104	0.027	−0.077
	CD20 on unswitched memory B cell	−0.121	0.037	0.001	0.886	−0.701	0.311	0.024	0.496	0.085	0.027	−0.058
	CD25 on IgD+ CD38dim B cell	−0.331	0.053	< 0.001	0.718	0.569	0.258	0.028	1.767	−0.189	0.027	0.216
	CD45RA on resting CD4 regulatory T cell	0.250	0.055	< 0.001	1.285	−0.183	0.090	0.041	0.833	−0.046	0.027	0.073
Gut bacterial pathway abundance (PWY.5177…glutaryl.CoA.degradation)	CD45RA+ CD8+ T cell Absolute Count	0.191	0.052	< 0.001	1.210	−1.255	0.504	0.013	0.285	−0.240	1.007	1.247
	CD4+CD8+ T cell Absolute Count	−0.120	0.053	0.023	0.887	0.657	0.320	0.040	1.930	−0.079	1.007	1.086
	CD20 on IgD− CD27− B cell	−0.312	0.068	< 0.001	0.732	−0.607	0.302	0.045	0.545	0.189	1.007	0.818
	CD25 on IgD+ CD38dim B cell	0.164	0.054	0.002	1.178	0.569	0.258	0.028	1.767	0.093	1.007	0.914
	CD25 on IgD− CD24− B cell	−0.174	0.054	0.001	0.840	1.325	0.507	0.009	3.761	−0.231	1.007	1.239
	CD45RA on resting CD4 regulatory T cell	0.155	0.056	0.005	1.168	−0.183	0.090	0.041	0.833	−0.028	1.007	1.036
Gut bacterial pathway abundance (PWY.5705…allantoin.degradation.to.glyoxylate.III)	CD45RA+ CD8+ T cell Absolute Count	0.096	0.012	< 0.001	1.101	−1.255	0.504	0.013	0.285	−0.121	0.501	0.622
	CD20 on IgD+ CD24+ B cell	−0.105	0.014	< 0.001	0.900	−0.781	0.372	0.036	0.458	0.082	0.501	0.419
	CD20 on unswitched memory B cell	−0.207	0.016	< 0.001	0.813	−0.701	0.311	0.024	0.496	0.145	0.501	0.357
	CD25 on IgD− CD24− B cell	−0.052	0.013	< 0.001	0.949	1.325	0.507	0.009	3.761	−0.069	0.501	0.570
	CD4 on CD39+ activated CD4 regulatory T cell	0.134	0.014	< 0.001	1.144	0.635	0.279	0.023	1.887	0.085	0.501	0.416
	CD45RA on resting CD4 regulatory T cell	−0.202	0.014	< 0.001	0.817	−0.183	0.090	0.041	0.833	0.037	0.501	0.464
Gut bacterial pathway abundance (PWY.5791…1.4.dihydroxy.2.naphthoate.biosynthesis.II…plants.)	CD45RA+ CD8+ T cell Absolute Count	0.154	0.020	< 0.001	1.167	−1.255	0.504	0.013	0.285	−0.194	0.171	0.364
	CD4+CD8+ T cell Absolute Count	0.158	0.022	< 0.001	1.171	0.657	0.320	0.040	1.930	0.104	0.171	0.067
	CD20 on IgD− CD27− B cell	−0.140	0.025	< 0.001	0.869	−0.607	0.302	0.045	0.545	0.085	0.171	0.085
	CD20 on unswitched memory B cell	0.047	0.022	0.037	1.048	−0.701	0.311	0.024	0.496	−0.033	0.171	0.203
	CD25 on IgD− CD24− B cell	−0.069	0.023	0.002	0.933	1.325	0.507	0.009	3.761	−0.092	0.171	0.263
	CD45RA on resting CD4 regulatory T cell	−0.310	0.028	< 0.001	0.733	−0.183	0.090	0.041	0.833	0.057	0.171	0.114
Gut bacterial pathway abundance (PWY.6151…S.adenosyl.L.methionine.cycle.I)	CD45RA+ CD8+ T cell Absolute Count	0.192	0.052	< 0.001	1.212	−1.255	0.504	0.013	0.285	−0.241	0.761	1.002
	CD25 on IgD+ CD38dim B cell	−0.144	0.064	0.025	0.866	0.569	0.258	0.028	1.767	−0.082	0.761	0.843
Gut bacterial pathway abundance (PWY.7211…superpathway.of.pyrimidine.deoxyribonucleotides.de.novo.biosynthesis)	CD25 on IgD− CD24− B cell	0.156	0.053	0.003	1.169	1.325	0.507	0.009	3.761	0.207	−0.730	−0.937
	CD4 on CD39+ activated CD4 regulatory T cell	0.114	0.058	0.049	1.121	0.635	0.279	0.023	1.887	0.072	−0.730	−0.802
Gut bacterial pathway abundance (PWY.724…superpathway.of.L.lysine…L.threonine.and.L.methionine.biosynthesis.II)	CD4+CD8+ T cell Absolute Count	−0.112	0.045	0.013	0.894	0.657	0.320	0.040	1.930	−0.074	1.112	1.185
	CD20 on IgD+ CD24+ B cell	−0.281	0.045	< 0.001	0.755	−0.781	0.372	0.036	0.458	0.220	1.112	0.892
	CD20 on unswitched memory B cell	−0.265	0.045	< 0.001	0.768	−0.701	0.311	0.024	0.496	0.185	1.112	0.926
	CD25 on IgD− CD24− B cell	0.124	0.063	0.050	1.132	1.325	0.507	0.009	3.761	0.164	1.112	0.947
Gut bacterial pathway abundance (PWY.7328…superpathway.of.UDP.glucose.derived.O.antigen.building.blocks.biosynthesis)	CD45RA+ CD8+ T cell Absolute Count	−0.079	0.028	0.005	0.924	−1.255	0.504	0.013	0.285	0.099	0.474	0.375
	CD4+CD8+ T cell Absolute Count	−0.106	0.032	0.001	0.899	0.657	0.320	0.040	1.930	−0.070	0.474	0.543
	CD20 on IgD− CD27− B cell	0.172	0.051	0.001	1.187	−0.607	0.302	0.045	0.545	−0.104	0.474	0.578
	CD20 on unswitched memory B cell	−0.153	0.037	< 0.001	0.858	−0.701	0.311	0.024	0.496	0.107	0.474	0.367
	CD25 on IgD+ CD38dim B cell	−0.068	0.032	0.035	0.934	0.569	0.258	0.028	1.767	−0.039	0.474	0.513
	CD25 on IgD− CD24− B cell	−0.270	0.033	< 0.001	0.764	1.325	0.507	0.009	3.761	−0.357	0.474	0.831
	CD4 on CD39+ activated CD4 regulatory T cell	0.121	0.035	0.001	1.129	0.635	0.279	0.023	1.887	0.077	0.474	0.397

Abbreviations: OR, odds ratio; SE, standard error.

*p* < 0.05 was considered as statistically significant.

## 4 Discussion

This study identified significant causal relationships between gut microbiota composition and congenital malformations of the nervous system, with specific immune cells playing mediating roles in these interactions. The results highlighted particular taxa and pathways within gut microbiota, such as the family *Eubacteriaceae* and the genus *Eubacterium*, which demonstrated a protective effect against nervous system malformations, as indicated by their negative associations with malformation risk. Conversely, other taxa, including *Eubacterium rectale* and *Bacteroides faecis*, showed a positive correlation with malformation risk. In examining the mediating role of immune system, nine immune cell indicators were identified as significant mediators. For example, CD20 expression on IgD^+^ CD24^+^ B cells exhibited a negative mediating effect, suggesting a protective mechanism against malformations, whereas CD25 expression on IgD^−^ CD24^−^ B cells showed a positive mediating effect, indicating an increased risk.

Our findings aligned with some existing research that connects gut microbial composition and immune responses to neonatal and developmental health. Several experimental studies have demonstrated that perturbations of maternal gut microbiota or administration of specific microbial metabolites during pregnancy alter offspring neurodevelopmental outcomes, supporting a causal link mediated by immune and metabolic changes. For example, maternal supplementation or depletion of butyrate producing bacteria alters systemic cytokine profiles and offspring neural inflammation in rodents (Lu et al. 2024; O'Riordan et al. 2025). Besides, human observational studies have reported associations between maternal microbiome composition and adverse neonatal outcomes. For instance, butyrate treatment in mothers conferred resistance in newborn mice to bile duct inflammation and injury, linked with specific microbial signatures like *Bacteroidetes* and *Clostridia* (Jee et al. 2022). This result complements our finding that certain *Clostridia* families (e.g., *Eubacteriaceae*) might offer protective effects against nervous system malformations, potentially through anti‐inflammatory mechanisms and metabolite regulation like glutamate. Furthermore, elevated colonization by pathogens such as *S. aureus* and *E. coli* in infants with cleft was identified, indicating that dysbiotic gut environments might worsen developmental outcomes (Qays and Jafar 2023). Similarly, the impact of maternal *Firmicutes* and *Bacteroidetes* on fetal brain lipid metabolism reinforced the potential for gut microbiota to influence neural development through the gut–brain axis (S. Wang et al. 2023). This intergenerational microbiota–metabolite correlation in NTDs highlighted a potential pathway through which specific microbial families in our study could modulate brain health and mitigate malformation risks. Additionally, the histidine and glutamate degradation pathways identified in our study as significant for congenital malformation risk were supported by research on their roles in congenital disorders (Chapman and Chen 2024; Duczyńska et al. 1990). These metabolic pathways were crucial in neurotransmission and neuroprotection, and their disruption might contribute to neurodevelopmental abnormalities.

Mechanistically, microbial metabolites and microbial‐associated molecular patterns (e.g., short‐chain fatty acids, amino acid catabolites, and lipopolysaccharide) may modulate maternal innate and adaptive immune responses, resulting in altered levels of cytokines, chemokines, and immune cell trafficking across the maternal–fetal interface. Such changes can influence placental nutrient and oxygen transport, fetal hematopoiesis and microglial maturation, which are critical for neural tube closure and early brain development (Loh et al. 2024; O'Riordan et al. 2025). Our identification of B cell and T cell markers (e.g., CD20, CD25, CD45RA, CD4/CD8 subsets) as mediators is consistent with these pathways, because B cells can regulate antibody and cytokine milieus and T cell phenotypes (including regulatory T cells) shape systemic and local immune tolerance critical to embryogenesis (He et al. 2025; Lund 2008). For CD20‐related findings, the negative mediating effects observed (e.g., CD20 on IgD^+^ CD24^+^ B cells) might reflect a protective role of certain B cell populations in maintaining maternal–fetal immune homeostasis. B cells could modulate cytokine production, produce natural antibodies, and influence placental immune responses, which together could lower inflammatory insults that disrupt neural tube closure (Lund 2008). Our MR results showing an inverse association between CD20‐related markers and malformation risk are therefore biologically plausible. Elevated CD25 expression during pregnancy might indicate heightened immune activation states which could promote proinflammatory pathways harmful to embryonic neural development (Huang et al. 2020). This mechanism could explain the positive mediation effect observed for CD25‐related markers. Furthermore, it was suggested that elevated CD45 levels in hematopoietic stem cells were linked to worsened outcomes in NTDs due to deteriorating hematopoietic signaling (Sahin Inan and Unver Saraydin 2019). CD45RA generally denotes naïve or specific T cell subsets. This supported our findings of the involvement of CD45 in neural malformations, possibly through its impact on hematopoiesis during development. Moreover, the role of CD8^+^ T cells, observed in congenital defects and developmental syndromes like DiGeorge syndrome (22q11.2DS), further supported our findings, as our study highlighted CD8 T cell markers as associated with higher malformation risks (Giardino et al. 2019; Yu et al. 2022). Alterations in CD8^+^ T cell subsets could reflect shifts in cytotoxic or effector cell balance that influence fetal hematopoiesis or local immune regulation at the maternal–fetal interface (Huang et al. 2025). This reflected the involvement of CD8 T cells in developmental pathways and highlighted their role in mediating the gut microbiota's effects on congenital malformations of the nervous system. Similarly, changes in CD4^+^CD8^+^ double‐positive counts might reflect thymic or developmental immune perturbations relevant to embryogenesis (Gordon and Manley 2011). Together, these findings corroborate the plausibility of our identification of gut–immune associations as contributors to congenital nervous system malformations in “gut–brain axis.”

The findings of this study suggested that specific gut microbiota and immune cells are associated with the risk of congenital malformations of the nervous system, which could have considerable clinical implications. For certain bacterial families that played a protective or risk role, targeted interventions to modulate gut microbiota composition, such as dietary adjustments, prebiotics, probiotics, or microbiota transplantation, might be considered to reduce malformation risks (Wolter et al. 2021). Similarly, if immune cells like CD20^+^ B cells or CD8 T cells were confirmed to influence neural development, immune‐modulatory therapies could be explored for their potential in minimizing neural malformation risks. These findings give a clue for future preventive strategies and clinical interventions aimed at reducing congenital malformation incidence by addressing gut–immune interactions.

This study had several limitations. The data source, predominantly European populations, may limit the generalizability of the findings to other populations. Additionally, MR relied on certain assumptions, such as the absence of pleiotropy and the strength of genetic variants as instrumental variables. The genetic variants chosen might not fully represent the exposures, leading to potential bias. Moreover, the causal inference in observational studies using genetic proxies posed challenges, as it may not account for all confounding factors, and the associations observed might not reflect direct causal relationships (Katikireddi et al. 2018).

To address these limitations, future studies should aim to validate these findings in larger and more diverse populations. They are expected to explore the dynamic relationship between gut microbiota, the immune system, and nervous system development through longitudinal studies that track these factors from pregnancy through early development. Additionally, investigating the specific mechanisms through which certain bacterial metabolites or immune cells impact neural development will provide deeper insights. Studies on how microbial metabolites regulate immune cell activity and influence brain development can help pinpoint precise intervention targets, further overcoming the gap between microbiota research and clinical applications in congenital malformation prevention.

## 5 Conclusions

In conclusion, this study underscored a significant causal relationship between gut microbiota and congenital malformations of the nervous system, mediated by specific immune cell types. These findings highlight the protective or risk‐bearing roles of certain bacterial families and immune markers, suggesting potential avenues for targeted interventions, such as microbiota modulation or immune therapies, to mitigate the risk of neural malformations. While promising, these results warranted further validation across diverse populations and deeper investigation into the precise microbial and immune mechanisms involved in neurodevelopmental pathways. Future research on gut–immune–neural interactions might offer valuable insights and opportunities for clinical advancements in reducing congenital malformation risks.

## Author Contributions

Conceptualization: Haokun Tian, Xiaolan Guo, Wensen Cao, Zhiqiang Zhu; Data curation: Haokun Tian, Jialu Yu, Aiyun Yang; Formal analysis: Haokun Tian, Xiaolan Guo; Funding acquisition: Jianhua Wang; Investigation: Xiaolan Guo, Xiuwei Wang, Shen Li, Xiaochun Xu; Methodology: Haokun Tian, Xiaolan Guo, Wensen Cao, Jialu Yu, Caihua Wang; Project administration: Haokun Tian, Jianhua Wang; Resources: Jialu Yu, Xiuwei Wang, Xiaochun Xu; Software: Haokun Tian, Xiuwei Wang; Supervision: Jialu Yu, Zhiqiang Zhu, Shen Li, Jianhua Wang; Validation: Xiaolan Guo, Zhen Guan; Visualization: Yuanfang Lu, Zhen Guan; Writing – original draft: Haokun Tian, Yuanfang Lu, Wensen Cao, Zhiqiang Zhu, Jianhua Wang, Caihua Wang; Writing – review & editing: Haokun Tian, Yuanfang Lu, Zhen Guan, Aiyun Yang, Jianhua Wang.

## Funding

This study was supported by Beijing Natural Science Foundation (7222016, 7244290), National Natural Science Foundation of China (U23A20420), and Research Foundation of Capital Institute of Pediatrics (CXYJ‐2021‐03).

## Ethics Statement

The authors have nothing to report.

## Consent

The authors have nothing to report.

## Conflicts of Interest

The authors declare no conflicts of interest.

## Supporting information




**Supporting table: 1** STROBE‐MR checklist of recommended items to address in reports of Mendelian randomization studies.

**Supporting table: 2** Reference table of gut microbiota and the pathway abundance indicators with renumbered index.

**Supporting table: 3** Summary data of Mendelian randomization using five different methods with 412 gut microbiota as exposures.

**Supporting table: 4** The results of heterogeneity using Cochran's *Q*‐test between SNPs for 19 gut microbiota indicators with causal associations and statistical significance.

**Supporting table: 5** The results of horizontal pleiotropy evaluation using MR‐Egger regression method.

**Supporting fig.1**: The results of heterogeneity among genetic variants detected using funnel plots.

## Data Availability

The original contributions presented in this study are included in the article/supporting information. Further inquiries can be directed to the corresponding author.
